# Companion dog acquisition and mental well-being: a community-based three-arm controlled study

**DOI:** 10.1186/s12889-019-7770-5

**Published:** 2019-11-05

**Authors:** Lauren Powell, Kate M. Edwards, Paul McGreevy, Adrian Bauman, Anthony Podberscek, Brendon Neilly, Catherine Sherrington, Emmanuel Stamatakis

**Affiliations:** 1 Charles Perkins Centre, Prevention Research Collaboration, Sydney School of Public Health, Faculty of Medicine and Health, University of Sydney, Sydney, NSW Australia; 2 Charles Perkins Centre, Faculty of Health Sciences, University of Sydney, Sydney, NSW Australia; 3 Sydney School of Veterinary Science, University of Sydney, Sydney, NSW Australia; 4 Charles Perkins Centre, Sydney School of Veterinary Science, University of Sydney, Sydney, NSW Australia; 5 Royal Society for the Prevention of Cruelty to Animals (RSPCA) NSW, Sydney, NSW Australia; 6 Institute for Musculoskeletal Health, Sydney School of Public Health, Faculty of Medicine and Health, University of Sydney, Sydney, NSW Australia

**Keywords:** Dog ownership, Companion dogs, Psychological health, Mental well-being, Mental health, Human-animal interactions, Depression, Anxiety, Affect, Loneliness

## Abstract

**Background:**

Dog ownership is suggested to improve mental well-being, although empirical evidence among community dog owners is limited. This study examined changes in human mental well-being following dog acquisition, including four measures: loneliness, positive and negative affect, and psychological distress.

**Methods:**

We conducted an eight-month controlled study involving three groups (*n* = 71): 17 acquired a dog within 1 month of baseline (dog acquisition); 29 delayed dog acquisition until study completion (lagged control); and 25 had no intentions of acquiring a dog (community control). All participants completed the UCLA Loneliness Scale (possible scores 0–60), Positive and Negative Affect Schedule and Kessler10 at baseline, three-months and eight-months. We used repeated measures ANCOVAs to analyse data with owner age and sex included as covariates. Post-hoc tests were performed for significant effects (*p* < 0.05).

**Results:**

There was a statistically significant group by time interaction for loneliness (*p* = 0.03), with an estimated reduction of 8.41 units (95% CI -16.57, − 0.26) from baseline to three-months and 7.12 (95% CI -12.55, − 1.69) from baseline to eight-months in the dog acquisition group. The group by time interaction for positive affect was also significant (*p* = 0.03), although there was no change in the dog acquisition group.

**Conclusions:**

Companion dog acquisition may reduce loneliness among community dog owners. Our study provides useful direction for future larger trials on the effects of dog ownership on human mental well-being.

**Trial registration:**

This trial was retrospectively registered on 5th July 2017 with the Australian New Zealand Clinical Trials Registry (ACTRN12617000967381).

## Introduction

The World Health Organization considers mental well-being as an integral component of health. A positive state of mental well-being allows individuals to recognise their potential, cope with normal stresses, work productively and contribute to society [1]. Many common stressors such as long working hours, poor economic conditions and low physical activity patterns can reduce mental well-being [2–4]. Further, mental illness is one of the leading contributors to the global burden of disease [5].

Dog ownership is common worldwide. For example, over 50% of households in the United States and 39% in Australia have dogs [6]. It has been suggested that dog ownership can improve human mental well-being through several possible pathways [7]. Dogs may provide their owners with social support and companionship [8, 9] and they may also act as catalysts for increased human social interactions [10–12]. Acute human–dog interactions have been shown to elicit positive hormonal effects including reduced cortisol concentrations, a biomarker of stress [13–15], and increased oxytocin concentrations [16–19]. Dog owners may also be more physically active than non-owners, as a result of dog-walking [20–24], with a well-established link between physical activity and positive mental well-being [3, 4].

Most research investigating mental well-being and human–dog interactions has examined the efficacy of animal-assisted therapies to improve psychological outcomes among institutionalised individuals, such as those living in nursing homes, or clinical populations with mental illness or chronic disease [25–30]. Among university students, dog-assisted interventions have also demonstrated that acute human–dog interactions have beneficial effects on measures of positive and negative affect [31, 32]. Longitudinal studies of dog ownership and mental well-being among community dwelling dog owners are rare. Only two studies, to date, have analysed the impact of companion animal acquisition on human physical and psychological health [33, 34], one of which reported positive results [33]. Both studies investigated a single indicator of mental well-being prior to and after pet acquisition, with a follow-up period of 6–10 months [33, 34]. A one-year prospective cohort study including *n =* 955 community-based older adults (≥65 years) has also been used to examine pet ownership and psychological well-being, documenting no association between ownership and overall satisfaction, happiness or perceived mental health [35]. The few cross-sectional correlate studies in the field have produced inconsistent findings [30]. For example, a survey of 1101 individuals residing in Perth, Australia suggested that dog owners are less lonely than non-owners [36], but other research found pet owners and non-owners do not differ in measures of loneliness [37] or psychological distress [37, 38].

The paucity of evidence and the conflicting results are partly attributable to a plethora of methodological challenges that are common in the field of human-animal interaction research [30]. The above cross-sectional studies [36–38] are limited as they compare existing dog or pet owners to non-owners and cannot rule-out reverse causation, i.e. the possibility that individuals who are interested in dog ownership experience better health prior to acquiring a dog [39]. Randomised controlled trials, in which human participants are randomly allocated to dog ownership, are not feasible in this field [40, 41]. Randomised assignment of dogs to uninterested members of the community would raise irreconcilable animal welfare concerns, such as the potential for neglect or inadequate care, including veterinary care. Dog ownership necessitates a substantial time and economic commitment which would also introduce human ethical concerns if uninterested individuals were allocated to dog ownership. As randomised controlled trials are not feasible, the strongest possible design for examining the impact of dog ownership on mental well-being may be controlled studies in which non-owners acquire a companion dog [41].

The aim of this controlled study was to examine potential changes in mental well-being among community dog owners following dog acquisition, using four common measures: loneliness, positive and negative affect, and psychological distress.

## Methods

### Study design

**Fig. 1:**
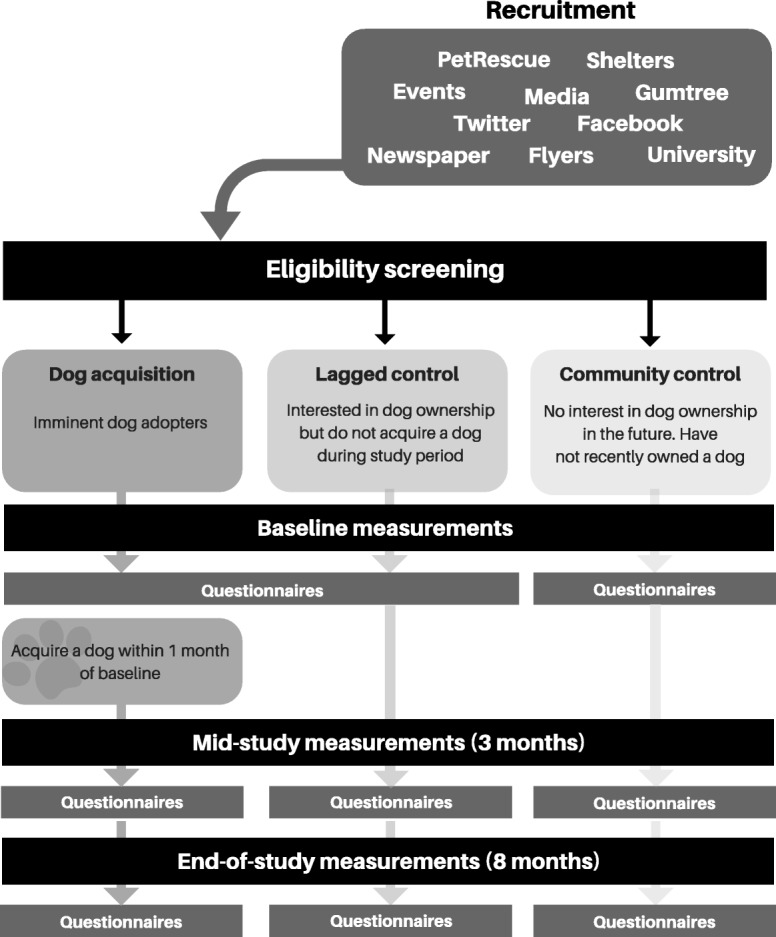
Study design and timeline

This study formed part of a larger three-arm controlled study in which the primary aim was to investigate the impact of community-based dog ownership on device-based and self-reported human physical activity (to be reported in detail elsewhere). Indicators of mental well-being were considered as secondary outcomes in the larger study but are the primary outcomes of the current manuscript. Upon completion of the baseline measurements, participants self-allocated to one of three treatment groups based on their dog ownership intentions: imminent dog adopters (“dog acquisition”); individuals interested in dog ownership but delayed from acquisition for the study duration (“lagged control”); and individuals who had no interest or plans to acquire a companion dog (“community control”) (Fig. 1). We included two control groups to account for possible differences in sociodemographic characteristics or health behaviours associated with an interest in dog ownership [42].

### Recruitment

Between April 2017 and September 2018, participants were recruited using media releases (TV, radio and newspaper); online adoption resources; focused events; University of Sydney communications; researcher attendance at animal welfare shelters; social media advertisements; and flyers distributed in the community.

Participants were eligible if they were aged 18 or over; resided within 60 km of the Sydney city centre, Australia; had an absence of physical limitations that could prevent walking; and did not currently own a dog or other furry pet (e.g. cat, rabbit) or plan to acquire one for the duration of the study. Individuals who had owned a dog in the 12 months prior to recruitment were excluded from the study. Participants in the dog acquisition group had to acquire a dog within 1 month of baseline measurements and be the main/joint carer of the dog. Additionally, the dog had to be free of veterinary conditions that would limit low intensity activities such as walking, and not have entered the last quintile of expected lifespan for their breed.

Participants were reimbursed for the time dedicated to participating in the study upon the completion of all measurements. Dog acquisition and lagged control group participants received a 12-month supply of routine dog medications, including vaccinations, internal and external parasite protection, and one veterinary appointment. Community control participants were offered $150 compensation.

Ethical approval was obtained from the University of Sydney Human Research Ethics Committee (2016/921) and Animal Ethics Committee (2017/1134). The study was registered with the Australian New Zealand Clinical Trials Registry (ACTRN12617000967381). All methods were performed in accordance with the relevant guidelines. All participants provided informed written consent.

### Questionnaires

We collected self-reported sociodemographic data including age, gender, level of education, companion animal ownership history, cardiometabolic health and lifestyle health habits, which are described here but will examined in detail elsewhere. Mental well-being questionnaires were administered three times over an eight-month period: at baseline, at three-months and at the end of the study.

#### Loneliness

The UCLA Loneliness Scale [43, 44] is a valid and reliable tool [45, 46] to measure loneliness and social isolation in community populations [34], including multiple Australian cohorts [47–49]. The 20-item questionnaire provides brief descriptions of feelings, such as ‘I am unhappy doing so many things alone.’ Participants reported how often they believed each description was indicative of them. The possible responses were never (0), rarely (1), sometimes (2) and often (3). Individual item scores were then added to provide a total score, with a possible range of 0 to 60 [44].

#### Positive and negative affect

The Positive and Negative Affect Schedule (PANAS) [50], and its short form [51] are valid and reliable tools to measure affect [52–54] and have been used in similar Australian community cohorts [55–57]. The Short PANAS, used in the current study, consists of 10 adjectives describing positive [5] or negative [5] emotions. Participants indicated the intensity of each emotion during the previous week with possible answers ranging from very slightly or not at all [1] to extremely [5]. Total positive and negative affect scores were calculated by adding the scores of each relevant item, with possible scores ranging from five to 25 [53].

#### Psychological distress

Kessler10 (K10) is a 10-item questionnaire which uses a Likert-type scale to measure psychological distress, specifically anxiety and depression, over the most recent 28-day period [58]. Participants were asked questions such as ‘During the last four weeks, about how often did you feel nervous?’ with 5 possible responses: none of the time (1), a little of the time (2), some of the time (3), most of the time (4) and all of the time (5). K10 has been used extensively across various populations and exhibits good psychometric qualities [59–61]. A total K10 score was calculated by summing the individual item scores, with a range of scores from 10 (no distress) to 50 (extreme distress) [62].

At mid- and end-of-study measurements, dog acquisition participants were asked an additional four questions regarding new social interactions they had experienced as a result of their dog (Additional file 1: Supplementary text) [63]. The questionnaire has demonstrated excellent reliability in comparable Australian cohorts [38]. We report these data as ancillary descriptive statistics.

### Statistical analysis

We used repeated measures ANCOVAs to examine the change in UCLA loneliness, positive and negative affect, and K10 scores following dog acquisition with owner age and sex included as covariates. In additional analyses, we also included education as a covariate. As the exposure was the same across the lagged control and community control groups (no dog acquisition), we conducted supplementary analyses comparing dog acquisition participants to a pooled group of control participants. To maximise use of available data, we also used repeated measures ANCOVAs to compare differences in questionnaire scores between baseline and three-month mid-study measurements where we included the five participants who did not complete the final eight-month study measurements. Post-hoc tests were performed for significant effects (*p* < 0.05). Partial Eta Squared (η_p_^2^) was determined as a measure of effect size. SPSS version 24 was used for all statistical analyses.

## Results

Ninety-six participants enrolled in the study and completed baseline data collection (26 in the dog acquisition group, 37 in the lagged control group and 33 in the community control group (Additional file 1: Figure S1). Seventy-one participants completed the study. Eight participants were excluded due to ineligibility following baseline measurements, such as moved outside the Sydney area (*n* = 3 dog acquisition, *n* = 2 lagged control, *n* = 3 community control). Six dog acquisition participants dropped out due to failure to acquire a dog (*n* = 3), unknown reasons (*n* = 2) or relinquishment (*n* = 1). Six lagged control participants dropped out for unknown reasons (*n* = 5) or withdrawing consent (*n* = 1). Five community control participants dropped out for unknown reasons (*n* = 3) or withdrawing consent (*n* = 2). There were no significant differences in baseline characteristics between participants who did not complete the study and the final sample, in terms of age, gender, education, smoking status, alcohol consumption, physical activity, sedentary behaviour patterns, loneliness, positive and negative affect, and psychological distress.

The baseline characteristics of participants who completed the study and were entered in the main analyses (*n* = 71) are presented in Table 1. There were statistically significant differences between the groups in terms of age (*p* = 0.01) and education (*p* = 0.02). Mean age was significantly higher in the community control group. The proportion of individuals who had completed university education was also greater in the lagged control and community control groups compared with the dog acquisition group. At baseline, loneliness (*p* = 0.66), positive affect (*p* = 0.39) and psychological distress (*p* = 0.16) were comparable between the groups. Negative affect was significantly greater in the dog acquisition group (*p* = 0.02).

**Table 1:** Baseline characteristics of the study sample by dog ownership status (*n* = 71)

Baseline characteristics	Dog ownership status		
	Dog acquisition (*n* = 17)	Lagged control (*n* = 29)	Community control (*n* = 25)
Age (years)	36.9 (10.6)	38.0 (13.6)	50.7 (18.4)
Gender (female %)	100	75.9	80.0
*Physical activity*			
Bouts of 10+ mins walking/week ^a^	11.5 (7.6)	8.3 (5.5)	8.9 (7.9)
Minutes spent walking/week ^a^	303.2 (277.7)	219.8 (192.4)	251.6 (202.7)
Time spent sedentary (hours/day) ^a^	7.7 (2.7)	7.8 (2.9)	7.4 (3.5)
*Smoking status*			
Current/Previous (%)	11.8	24.1	32.0
Never (%)	88.2	75.9	68.0
*Alcohol consumption*			
1 or more days/week	70.6	55.2	56.0
Less than once per week	29.4	44.8	44.0
*Education* ^b^			
Trade certificate/diploma or less (%)	47.1	17.2	12.0
Bachelor’s or post graduate degree (%)	52.9	82.8	88.0
Previous dog ownership (%)	52.9	65.5	44.0
*Dogs as social catalysts* ^c^			
Got to know people in their neighbourhood (%)	82.4	N/A	N/A
Considered this person a friend (%)	35.3	N/A	N/A
Could ask for information (%)	76.5	N/A	N/A

Data are presented as mean (standard deviation) unless indicated otherwise

^a^Based on participant’s self-reported physical activity and sedentary behaviour patterns

^b^Highest level of education completed

^c^Based on responses to dogs as social catalysts questionnaire at end-of-study measurements

Approximately half of participants in the dog acquisition group (*n* = 9) had previously owned a dog, most as the primary or co-carer (*n* = 6). Many dog adopters reported new social interaction following dog acquisition with 82.4% of dog acquisition participants reporting they met people in their neighbourhood because of their dog, and 76.5% considering such people as sources of advice (Table 1). A smaller proportion of dog adopters (35.3%) considered the new social connection as a friend.

### Impact of dog acquisition on indicators of mental well-being

**Fig. 2:**
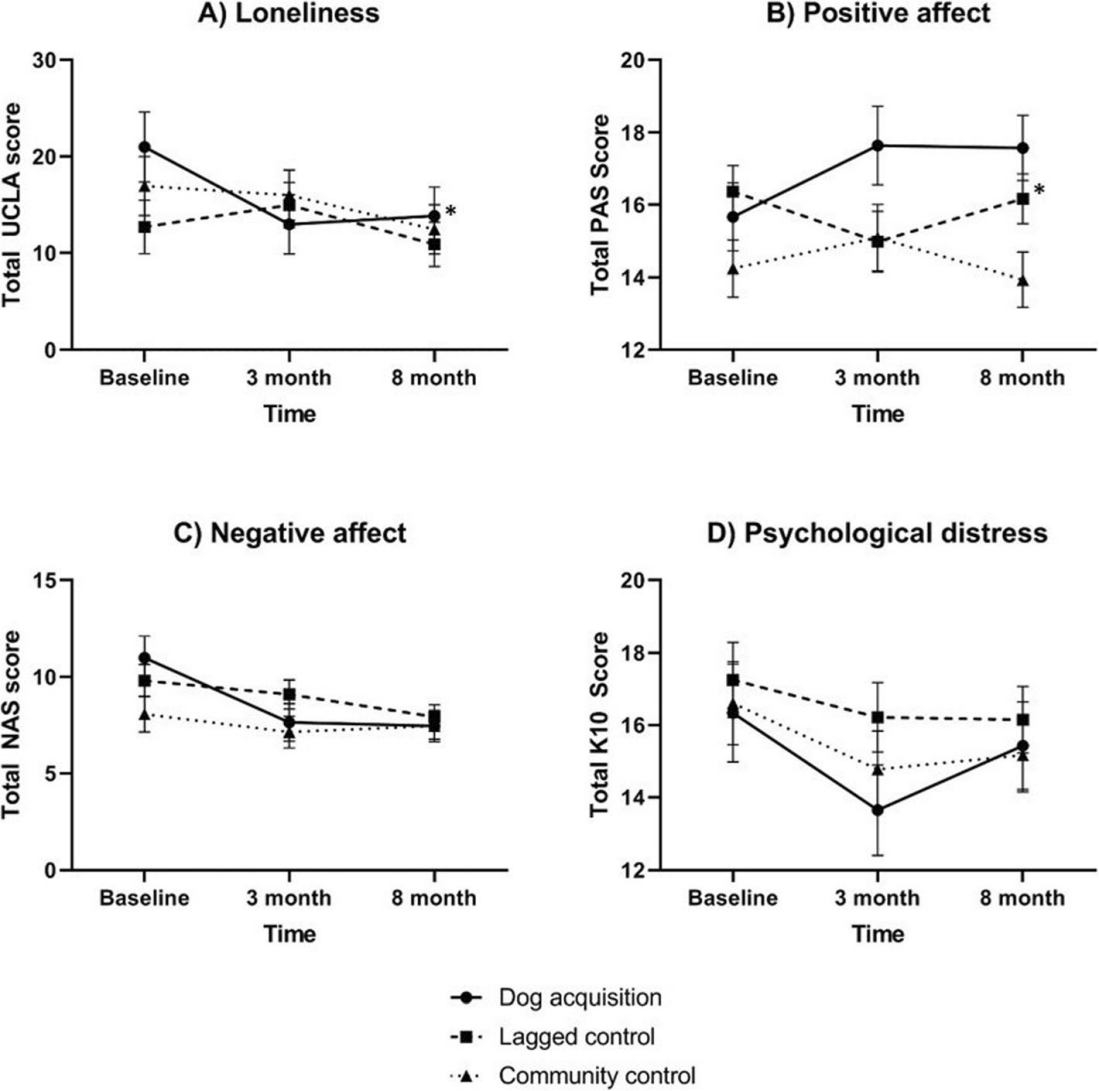
Estimated marginal means and the standard error of the mean for questionnaire scores by dog ownership status, adjusted for age and sex. **a** Loneliness. Possible UCLA loneliness scores range from 0 to 60. **b** Positive affect. Possible Positive Affect Schedule (PAS) scores range from 5 to 25. **c** Negative affect. Possible Negative Affect Schedule (NAS) scores range from 5 to 25. **d** Psychological distress. Possible Kessler10 (K10) scores range from 10 to 50. *Denotes a statistically significant group by time interaction in repeated measures ANCOVA (*p* < 0.05)

Figure 2 presents the estimated marginal mean scores (adjusted for age and sex) for loneliness, positive and negative affect, and psychological distress by study group (*n* = 71).

#### Loneliness

Repeated measures ANCOVA showed a statistically significant group*time interaction for loneliness (*F*(4,132) = 2.68, *p* = 0.03, *η*_*p*_^*2*^ = 0.08). The dog acquisition group displayed an estimated mean reduction of 8.41 units (95% confidence intervals (CI) -16.57, − 0.26, *p* = 0.04) from baseline to mid-study and 7.12 units (95% CI -12.55, − 1.69, *p* = 0.01) from baseline to end-of-study.

#### Positive and negative affect

We observed a significant group*time interaction in repeated measures ANCOVA for positive affect (*F*(4,132) = 2.75, *p* = 0.03, *η*_*p*_^*2*^ = 0.08). Among the lagged control group, post-hoc tests estimated a mean reduction of 1.24 units (95% CI -2.33, − 0.15, *p* = 0.03) in the positive affect scale from baseline to mid-study. There were no significant differences in the dog acquisition (*p* = 0.15) or control groups (*p* = 0.43). For negative affect, the group*time interaction was *F*(4,132) = 2.39, *p* = 0.05, *η*_*p*_^*2*^ = 0.07.

#### Psychological distress

There were no statistically significant group by time effects for psychological distress (*F*(4,132) = 0.61, *p* = 0.66, *η*_*p*_^*2*^ = 0.02).

### Pooled control group analyses

Additional file 1: Figure S2 displays the estimated marginal means (adjusted for age and sex) for loneliness, positive and negative affect, and psychological distress for the dog acquisition and pooled control groups (*n* = 71). Repeated measures ANCOVA analyses were performed for each outcome (2 group × 3 time points).

The group*time interaction for loneliness was statistically significant (*F*(2,134) = 4.70, *p* = 0.01, *η*_*p*_^*2*^ = 0.07). Mirroring the results of the primary analysis, the dog acquisition group displayed a statistically significant reduction of 8.41 units (95% CI -16.57, − 0.26, *p* = 0.04) from baseline to mid-study and 7.12 units (95% CI -12.55, − 1.69, *p* = 0.01) from baseline to end-of-study. In contrast to the primary analysis, loneliness scores were also significantly reduced in the combined control group, with a mean reduction of 3.06 units (95% CI -5.25, − 0.86, *p* = 0.01) between baseline and end-of-study measurements. For positive affect, the group*time interaction was non-significant (*F*(2,134) = 2.71, *p* = 0.07, *η*_*p*_^*2*^ = 0.04). For negative affect, there was a statistically significant group*time interaction (*F*(2,134) = 3.60, *p* = 0.03, *η*_*p*_^*2*^ = 0.05). Contrary to the primary results, we observed a statistically significant reduction in the dog acquisition group of 3.59 units (95% CI -6.31, − 0.87, *p* = 0.01) in the negative affect scale from baseline to mid-study and 3.53 units (95% CI -5.51, − 1.55, *p* = 0.002) from baseline to end-of-study. The combined control group also displayed a significant reduction between baseline and the end-of-study measurements (estimated mean change − 1.3, 95% CI -2.19, − 0.40, *p* = 0.01). In agreement with the primary analyses, the group*time interaction for psychological distress was not statistically significant (*F*(2,134) = 1.03, *p* = 0.36, *η*_*p*_^*2*^ = 0.02).

### Baseline to three-month analyses

Supplementary repeated measures ANCOVA analyses including all participants with valid data at baseline and mid-study measures (*n* = 76) produced similar results to the primary analyses. We observed a significant group*time interaction in loneliness (*F* (2, 71)=4.66, *p* = 0.01, *η*_*p*_^*2*^ = 0.12). Among the dog acquisition group, there was a mean reduction of 8.4 (95% CI -15.66, − 1.18, *p* = 0.03) units in the loneliness scale. There was also a statistically significant group*time interaction in positive affect (*F* (2, 71)=4.09, *p* = 0.02, *η*_*p*_^*2*^ = 0.10), with the lagged control group displaying a decrease (− 1.19, 95% CI -2.30, − 0.09, *p* = 0.04). Among the dog acquisition group, positive affect was not significantly different. The group* time interactions for negative affect and psychological distress were not statistically significant (*F* (2, 71)=1.86, *p* = 0.16, *η*_*p*_^*2*^ = 0.05 and *F* (2, 71)=0.75, *p* = 0.78, *η*_*p*_^*2*^ = 0.02, respectively).

### Additional adjustment for education

Repeated measures ANCOVA analyses with adjustment for owner age, gender and education (*n* = 71) produced null findings for all four scales. The additional education adjustment nullified the association between dog acquisition and loneliness, presenting a group*time interaction of *F*(4,130) = 1.85, *p* = 0.12, *η*_*p*_^*2*^ = 0.05. The group*time interactions for positive affect and negative affect were *F*(4,130) = 2.41, *p* = 0.05, *η*_*p*_^*2*^ = 0.07 and *F*(4,130) = 2.28, *p* = 0.06, *η*_*p*_^*2*^ = 0.07, respectively. For psychological distress, the group*time interaction was *F*(4,130) = 0.65, *p* = 0.63, *η*_*p*_^*2*^ = 0.02.

## Discussion

The aim of this study was to examine changes in mental well-being following dog acquisition, including four measures: loneliness, positive and negative affect, and psychological distress. This controlled study provides some of the first longitudinal evidence that dog acquisition may reduce loneliness among community-dwelling dog owners. Following dog acquisition, we observed a moderate reduction [64] in loneliness within 3 months, with the observation persisting until the end of the study. The significant difference in loneliness was also apparent in supplementary analyses including the pooled control group. A possible explanation for our findings is that human–dog interactions elicit acute positive effects on mood [31, 32, 65], and the regular occurrence of these interactions, as seen in dog ownership, produces long-term improvements. Indeed, research investigating the efficacy of canine interactions in reducing psychological distress in university students [31, 32] and preadolescents [65] has found brief human–dog interactions can acutely improve positive affect and reduce negative affect. Considering the association between loneliness and negative mood [66], it is plausible that the potential mood enhancing effects of regular human–dog interactions may reduce loneliness. Similarly, cross-sectional research has shown that support from a companion animal mediates the relationship between loneliness and negative mood in older women [67]. Another possible explanation is that dog ownership increases human social interaction, thereby improving the social well-being of dog owners and reducing their loneliness. Dogs may act as catalysts for social interaction [10, 11, 68, 69]. An ancillary finding in our study to support this explanation was that most dog owners had met people in their neighbourhood because of their dog and some even considered such people as potential sources of advice. Accordingly, a preliminary investigation of the possible mediating role of human social interaction in alleviating loneliness has shown dog walkers who conversed with others during their walks reported lower levels of loneliness compared with dog walkers who did not converse with others [70]. Our results are discordant with a previous quasi-experimental study that investigated companion animal acquisition and loneliness using the UCLA scale [34]. In their sample of 59 adults, 16 of whom had acquired a dog by the end of the study, Gilbey, McNicholas [34] found no significant differences in loneliness following cat or dog acquisition.

In the supplementary analyses including adjustment for education as an indicator of socioeconomic status (SES), the association between dog ownership and loneliness was nullified. Current literature indicates an increased risk of mental illness with low SES [71–73]. In the present study, SES may also have influenced the impact of dog acquisition on loneliness. For example, low SES individuals may have experienced reduced social support [74, 75] at baseline and as such, gained greater benefit from the social support and companionship provided by dogs. However, we did not collect data on participants’ social support, which would have aided this interpretation. In the supplementary analyses including the pooled control groups, there was also a reduction in loneliness among this group. The differences in self-reported mental well-being among the pooled control group may be the result of study participation, whereby individuals alter their responses or behaviour due to their awareness of being observed [76, 77].

We did not find evidence that dog acquisition influenced positive affect although, there was a significant difference in the lagged control group, with a moderate reduction [64] in positive affect at 3 months. The difference in positive affect did not persist at 8 months or in supplementary analyses including the pooled control group. Considering negative affect, we found evidence that dog acquisition was associated with a moderate reduction [64] among dog adopters. Although the results did not reach statistical significance in the primary analysis, we observed a significant reduction in the dog acquisition group when we pooled the control groups. Similarly to loneliness, the reduction occurred rapidly within 3 months and persisted until the end of the study. The mood enhancing effects of acute human–dog interactions, detailed above, may improve chronic measures of affect through the occurrence of regular acute human–dog interactions as seen in dog ownership [31, 32, 65]. We also observed a reduction in negative affect in the lagged control group, albeit to a lesser extent than the dog acquisition group, which may be the result of study participation effects [78], as described above.

We did not find evidence that dog acquisition significantly affects psychological distress. Our findings are congruent with prior cross-sectional studies that found companion animal ownership was not associated with symptoms of anxiety or depression [37, 38]. Conversely, the only comparable observational study that has investigated dog acquisition and symptoms of psychological distress reported a significant reduction in General Health Questionnaire scores among a sample of 47 dog adopters (total *n* = 71) [33]. Other cross-sectional studies have suggested that pet owners report greater depressive symptoms [55]. Overall, the contradictory results highlight the need for further research in dog ownership and mental well-being.

One of the strengths of our controlled study is the longitudinal design. To our knowledge, only two studies to date have used similar designs to investigate dog ownership and human mental well-being [33, 34], one of which was conducted almost three decades ago [33]. Another strength is the use of a broad range of measures to capture mental well-being. There are also several limitations of the study which necessitate cautious interpretation of our findings. Firstly, there is a lack of randomisation of dog ownership, which is not feasible for this exposure. There is also a lack of allocation concealment and blinding. As a result, selection bias may have occurred due to participants’ self-selection to their group. We also found differences between the treatment groups in terms of age and education. To reduce the possible impacts of these imbalances, we investigated changes in mental well-being over time and adjusted for both age and education. However, it must be noted that the adjustment for education nullified the results. There was also a significant difference at baseline between the groups in negative affect. The small sample size of the dog acquisition group suggests this analysis may have been statistically underpowered, which could have contributed to the instability in our results. For example, by altering the grouping of participants, such as pooling the control groups in the supplementary analyses, we found some results were inconsistent with the primary analyses. The pooling of control participants may also have introduced response bias because participants who expressed an interest in dog ownership may be inherently different from those with no ownership intentions. Finally, the absence of males in the dog acquisition group may limit the generalisability of our findings.

## Conclusions

In this sample of Australian urban dog owners, acquisition of a dog was associated with a reduction in loneliness within three months, with the observation persisting to the end of the study. Our results are suggestive of a relatively rapid, positive impact of dog acquisition on some indicators of human mental well-being. Our study provides preliminary, albeit unique, insights to inform future larger controlled studies on the relationship between dog ownership and human mental health.

## Supplementary information

**Additional file 1: Supplementary text.** Dogs as catalysts for new social interactions. **Figure S1.** CONSORT flow diagram modified for non-randomized trial design. **Figure S2.** Estimated marginal means adjusted for age and sex with standard error of the mean for questionnaire scores for dog acquisition and the pooled control group. **A)** Loneliness. Possible UCLA loneliness scores range from 0 to 60. **B)** Positive affect. Possible Positive Affect Schedule (PAS) scores range from 5 to 25. **C)** Negative affect. Possible Negative Affect Schedule (NAS) scores range from 5 to 25. **D)** Psychological distress. Possible Kessler10 (K10) scores range from 10 to 50. *Denotes a statistically significant group by time interaction in repeated measures ANCOVA (*p* < 0.05).

## Data Availability

The datasets generated during the current study are not publicly available due to the requirements of the ethical approval but are available from the corresponding author on reasonable request.

## Abbreviations

CI: Confidence intervals
K10: Kessler 10
PANAS: Positive and Negative Affect Schedule
