# Supplementary material for: Companion dog acquisition and mental well-being: a community-based three-arm controlled study
Source: BMC Public Health. 2019 Nov 5;19:1428. doi: 10.1186/s12889-019-7770-5 (PMC6829935; doi:10.1186/s12889-019-7770-5)
Supplement: Supplementary file 1 — Additional file 1: Supplementary text. Dogs as catalysts for new social interactions. Figure S1. CONSORT flow diagram modified for non-randomized trial design. Figure S2. Estimated marginal means adjusted for age and sex with standard error of the mean for questionnaire scores for dog acquisition and the pooled control group. A) Loneliness. Possible UCLA loneliness scores range from 0 to 60. B) Positive affect. Possible Positive Affect Schedule (PAS) scores range from 5 to 25. C) Negative affect. Possible Negative Affect Schedule (NAS) scores range from 5 to 25. D) Psychological distress. Possible Kessler10 (K10) scores range from 10 to 50. *Denotes a statistically significant group by time interaction in repeated measures ANCOVA (p < 0.05). [file 12889_2019_7770_MOESM1_ESM.docx]

**Supplementary text.** Dogs as catalysts for new social interactions

Dog owners were asked “Have you got to know people in your neighbour as a result of your dog (for example, through walking your dog or talking to your neighbours about your dog)?” This item was then adapted slightly to ask participants “Have you got to know people elsewhere as a result of your dog (for example, through walking your dog on the beach or in a park that’s not in your neighbourhood)?”

Dog owners who answered “yes” to either of these questions, were then asked “Do you regard any of the people you have met through your dog as a friend (more than just an acquaintance)?” and “Have you met anyone through your dog who you could:

- talk with about something that was worrying you such as a work or family issue?”
- ask for information such as, if they could recommend a tradesperson or restaurant?”
- ask for advice?”
- ask to borrow something (such as a book or tool), or ask a favour (such as collect mail) or ask for practical help such as getting a ride?”


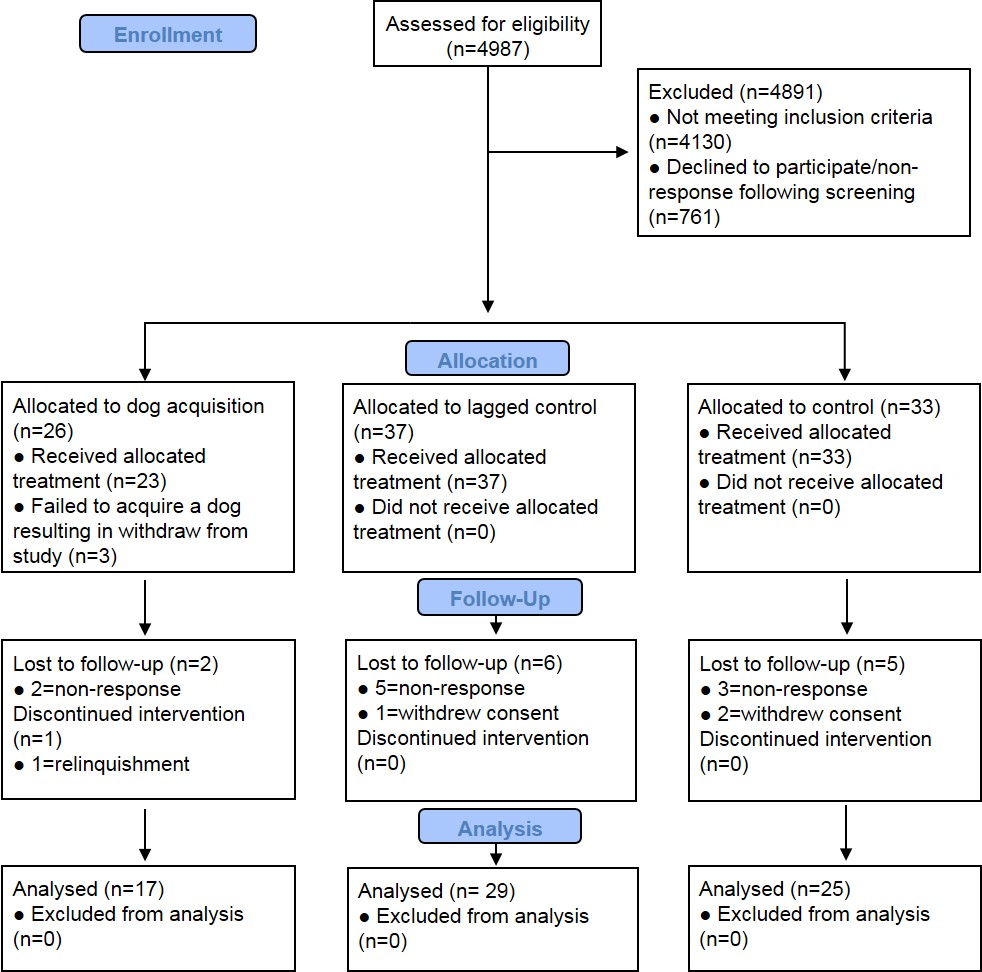


**Supplementary Figure 1.** CONSORT flow diagram modified for non-randomized trial design.

**
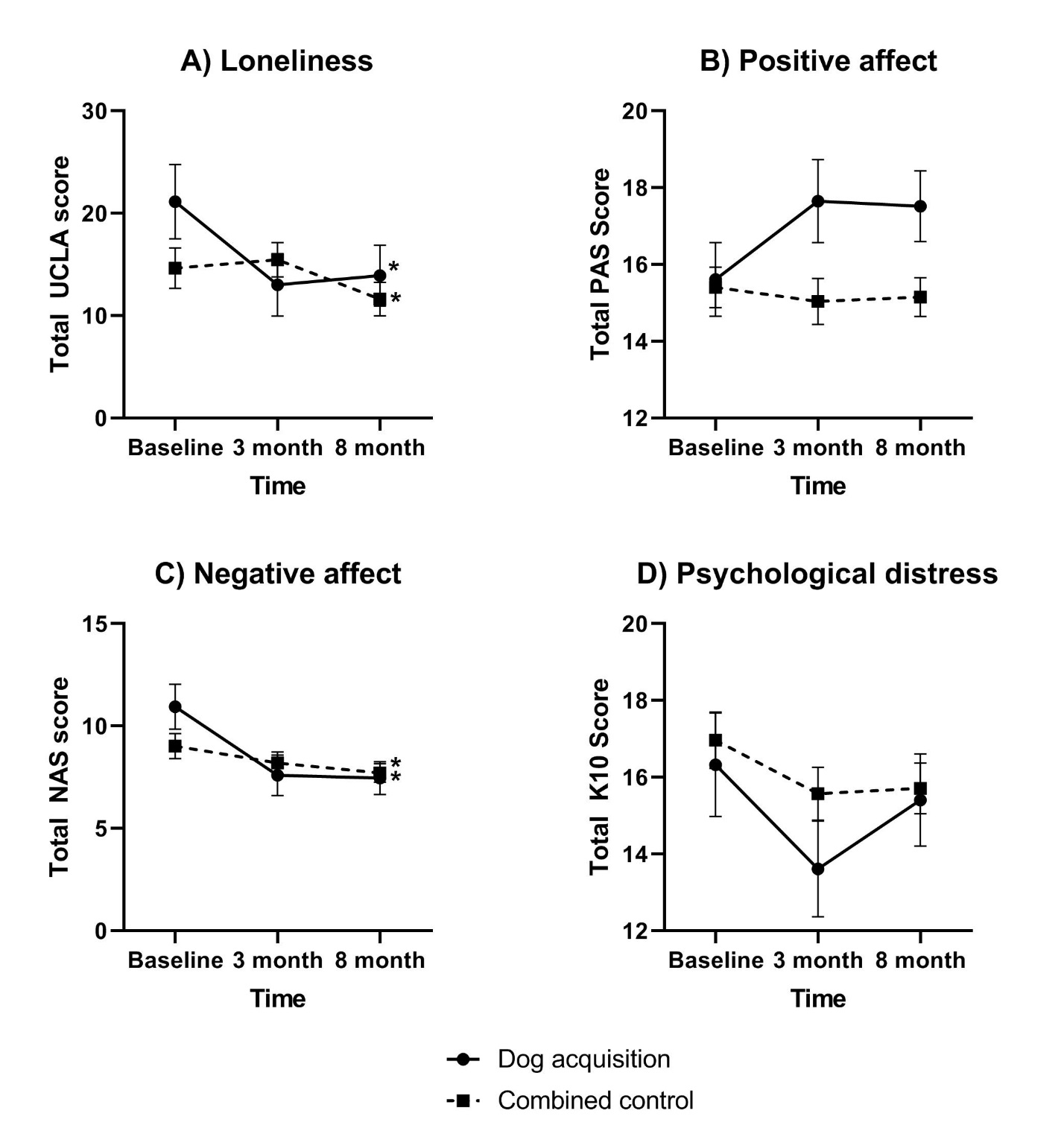
Supplementary Figure 2.** Estimated marginal means adjusted for age and sex with standard error of the mean for questionnaire scores for dog acquisition and the pooled control group. **A)** Loneliness. Possible UCLA loneliness scores range from 0 to 60. **B)** Positive affect. Possible Positive Affect Schedule (PAS) scores range from 5 to 25. **C)** Negative affect. Possible Negative Affect Schedule (NAS) scores range from 5 to 25. **D)** Psychological distress. Possible Kessler10 (K10) scores range from 10 to 50. *Denotes a statistically significant group by time interaction in repeated measures ANCOVA (*p*<0.05).
